# The Effects of Microplastics on Musculoskeletal Disorder; A Narrative Review

**DOI:** 10.1007/s12178-024-09932-9

**Published:** 2024-11-22

**Authors:** Hiroyori Fusagawa, Alex Youn, Elyse Wilkerson, Nirav Pandya, Brian T. Feeley

**Affiliations:** 1 Department of Orthopaedic Surgery, University of California-San Francisco, 1500 Owens Street, San Francisco, CA 94158 USA; 2 School of Medicine, University of California-San Francisco, 505 Parnassus Ave MU 320W, San Francisco, CA 94143 USA; 3 Department of Biomedical Engineering, College of Engineering, Carnegie Mellon University, 5000 Forbes Avenue, Pittsburgh, PA 15213 USA; 4 Department of Chemical Engineering, College of Engineering, Carnegie Mellon University, 5000 Forbes Avenue, Pittsburgh, PA 15213 USA

**Keywords:** Microplastics, Musculoskeletal disorder, Bone, Muscle

## Abstract

**Purpose of Review:**

The physical health impacts of microplastics have received increasing attention in recent years. However, limited data impedes a full understanding of the internal exposure to microplastics, especially concerning the musculoskeletal system. The purpose of this review is to summarize the recent literature regarding the effects of microplastics on the musculoskeletal system.

**Recent Findings:**

Microplastics have been shown to cause abnormal endochondral ossification and disrupt the normal function of pre-osteoblasts, osteocyte-like cells, and pre-osteoclasts through gene mutations, endoplasmic reticulum stress induction, and reduced autophagosome formation in bone growth areas. Although there are few reports on their effects on muscle, it has been noted that microplastics inhibit energy and lipid metabolism, decrease type I muscle fiber density, impair muscle angiogenesis, cause muscle atrophy, and increase lipid deposition.

**Summary:**

Only a few recent studies have shown that microplastics interfere with the normal function of bone growth-related cells and reduce muscle mass and quality. This review underscores the need for further research into other parts of the musculoskeletal system and studies using human tissues at the disease level.

## Introduction

Plastic was invented in the mid-nineteenth century, but the plastic boom in the mid-twentieth century has led to over nine billion metric tons of plastic that have been manufactured [1]. There has been a 19,000% increase in global plastic production between 1950 and 2015 [2]. As such, plastic has become an everyday commodity used in a majority of industries due to its affordability and convenience. Microplastics (MPs) are plastic by-products produced in the making of plastic and due to plastic degradation [3]. In recent years, it has been reported that MPs affect several organ systems in the body [4], a concern that all healthcare providers, including orthopedic surgeons specializing in the musculoskeletal system, cannot overlook. There are new concerns as to the extent to which MPs are related to orthopedic diseases encountered in daily practice, and the extent to which MPs may affect limb growth. It is critical to understand how MPs impact the development and homeostasis of musculoskeletal function. Therefore, the purpose of this review is to summarize the known effects of MPs on the musculoskeletal system and to identify areas of research on microplastics that need further exploration in the future.

## What are Microplastics?

Microplastics are pieces of plastic debris in the environment that are smaller than 5 mm and nano plastics are smaller than 1 μm [5]. They can be grouped into primary and secondary groups and come from several different sources. Primary MPs are specifically manufactured to be small, often less than 5 mm in diameter [3]. These MPs are found in skincare products, plastic pellets, and industrial abrasives. Secondary MPs are created when larger plastic items break down. There are many forms of plastic, such as Polyethylene (PE) and Polypropylene (PP), Polystyrene (PS), Polycarbonate, Polyurethane, Polyamide, Polyethylene terephthalate (PET), and Polyvinyl Chloride (PVC), that all break down differently in the environment [6]. PE and PP (e.g. margarine tubs, yoghurt cups, food trays, packaging film), PVC (e.g., window shutters, water pipes, upholstery), PS (e.g., packages, car parts, Styrofoam), and PET (e.g., beverage bottles, polyester fabrics) account for 90% of total plastic production worldwide. Thus, it is extremely difficult to live completely free of MPs in modern life.

Plastics are designed to resist degradation yet are still broken down by various methods before they are ingested by people. Biodegradation by microorganisms requires centuries to decompose plastics [7]. In addition to these degradation processes in the natural environment, recent studies have shown more cautionary degradation processes in methods more familiar to our daily lives. Hussain et al. found that microwave heating caused the higher release of MPs and nanoplastics (NPs) into food from plastic containers (4.22 million MPs from an area of 1 cm^2^ in just 3 min of microwave heating) compared to other usage scenarios, such as refrigeration or room-temperature storage [8]. A study comparing single-use plastic bottles to recyclable plastic bottles found that repeated use of the same bottle induces wear and tear and releases MPs that can enter the body [9]. It is also shown that repeated physical friction of everyday items (e.g., car tires, toys, cleaning agents) can cause plastics to break down into MPs [10].

After plastics have decomposed into MPs and NPs, they can be ingested by humans through the airway, skin, and diet. The primary route of MPs entering body is through food, especially seafood, given the abundance of MPs in the marine environment [11]. A recent meta-analysis suggested that human ingest 0.1–5 g of MPs weekly through consumption of everyday foods [12]. MPs are spread through wind currents and are inhaled nasally and orally, which has been linked to respiratory and cardiovascular diseases (see General health effects section below) [3]. Although ingestion through the skin is less common, MPs can penetrate through human skin with nanoscale particles which are less than 100 nm in size [13].

## General Health Effects

**Fig. 1:**
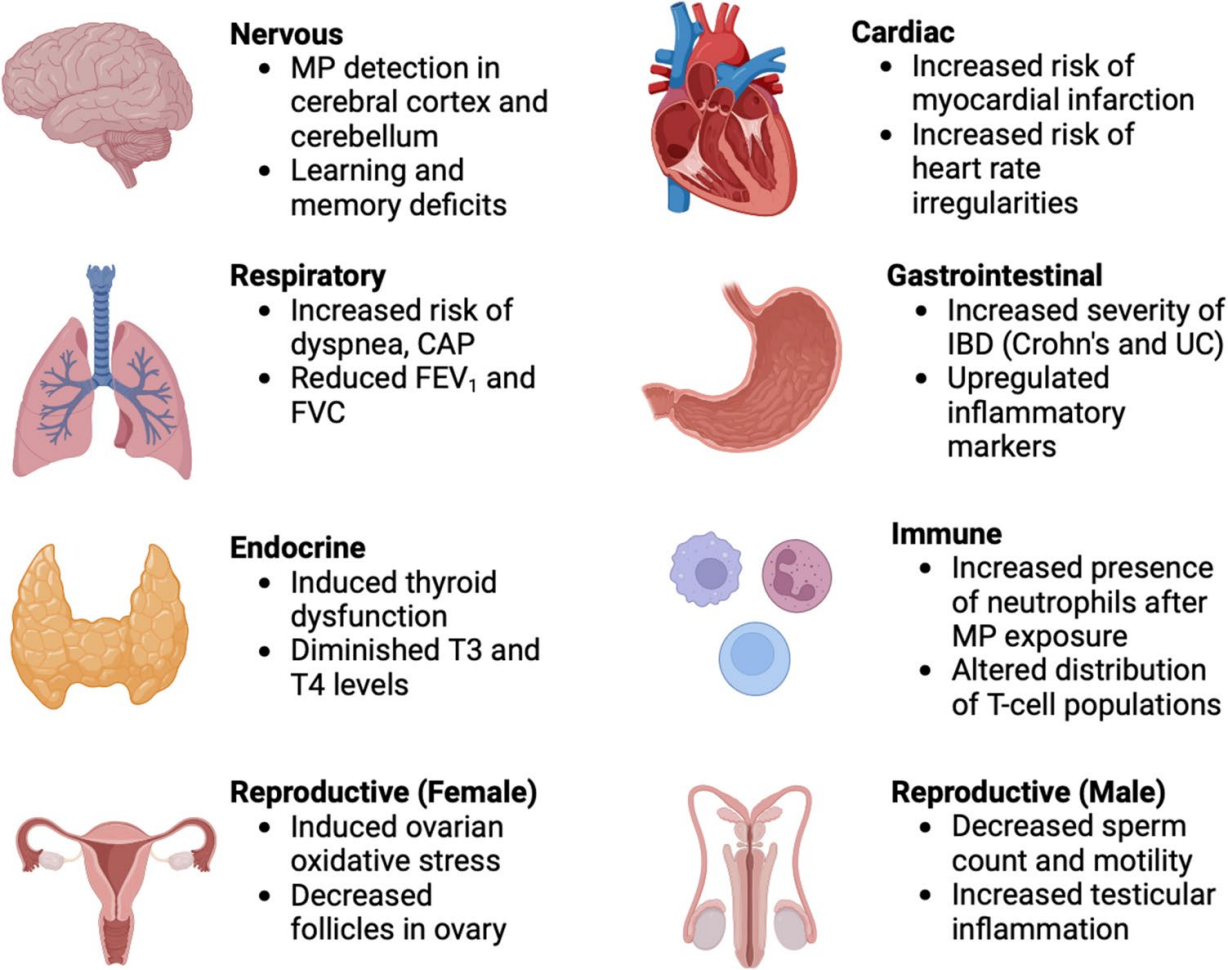
General health effects of microplastics in human and mouse models, including the nervous [27, 74], cardiac [14, 16], respirator [21, 22], gastrointestinal [25, 26], endocrine [75], immune [76], and female [77] and male reproductive systems [29, 78]. MP = Microplastic; CAP = community-acquired pneumonia; FEV_1_ = forced expiratory volume in one second; FVC = flow-controlled ventilation; T3 = triiodothyronine; T4 = thyroxine; IBD = inflammatory bowel disease; UC = ulcerative colitis

After entering the body through the various pathways outlined above, MPs have been associated with a wide range of effects throughout the human body (Fig. 1). First, MPs have been implicated in increased risk of cardiovascular disease. Marfella et al. followed 304 patients who were undergoing carotid endarterectomy for asymptomatic carotid artery disease and found that patients with carotid artery plaques positive for the presence of micronanoplastics were at a significantly higher composite risk for myocardial infarction, stroke, or death of any kind than patients with plaques without micronanoplastics [14]. Similarly, Yang et al., in a study consisting of 101 participants, found that patients with acute coronary syndrome and acute myocardial infarction were found to have significantly higher microplastic concentrations than controls [15]. Furthermore, albeit not in human models, MPs have been linked to abnormal heart rates [16], decreased ventricular contraction frequency [17], and pericardial edema [18].

Moreover, MPs have been found in the presence of human lung tissue [19], and MP exposure, especially in their commonly inhaled mode of transmission, has been associated with various forms of respiratory toxicity. For instance, synthetic textile workers, with chronic exposure to high concentrations of airborne microplastics, have been shown to have increased risk for developing dyspnea and sinusitis, compared to nonexposed controls [20]. Ng et al. showed that polyvinyl chloride (PVC) workers were found to have reduced forced expiratory volume and flow-controlled ventilation measurements, in addition to higher prevalence of chest tightness and wheezing, compared to nonexposed controls [21]. Chen et al. measured the level of MPs in bronchoalveolar lavage fluid in pediatric patients in China diagnosed with community-acquired pneumonia (CAP) and found that MP concentration was significantly higher in severe CAP patients than in non-severe patients, further suggesting the danger of MPs as an environmental pollutant [22].

The effects of MPs on the gastrointestinal system have also been widely explored. Multiple studies have detected the presence of microplastics in human feces [23, 24]; in fact, concentration of MP exposure has been shown to be positively correlated to severity of inflammatory bowel disease, specifically Crohn’s disease and ulcerative colitis [25]. These findings are substantiated by studies showing that MPs are potential triggers for intestinal dysbiosis, as evidenced by enrichment of Chlamydia, Firmicutes, and Proteobacteria, along with upregulating pro-inflammatory markers, including IL-1α, IL-1β, TNF-α, IFN-γ and IL-6 [26].

The deleterious effects of MPs are also beginning to be reported elsewhere in the body; notably in the nervous (e.g., learning and memory deficits [27]), endocrine (e.g., thyroid dysfunction [28]), and reproductive systems (e.g., decreased sperm motility [29]) in animal models, but the involvement of MPs using human disease groups has yet to be fully explored [13].

## General Cellular Effects

### Nucleus/Chromosomes

One of the primary ways MPs impact cellular function is through inducing DNA damage. Martin-Folgar et al. assessed the effects of polystyrene nanoplastics (PSNPs) on hNS1, a cell line model of human neural stem cells derived from human fetal forebrain [30]. They found that *gadd45α* and *rad51*—genes located in human neural stem cells and associated with DNA repair mechanisms of nucleotide excision and double-strand breaks, respectively—were elevated in a dose-dependent manner after exposure to PSNPs for four days. Expression of *xrcc1*, in contrast, was decreased. Notably, the deletion of this gene has previously been linked to neuropathologies, particularly hippocampal abnormalities and cerebellar interneuron loss [31]. These results suggest the potential impact that PSNPs can have on DNA repair mechanisms and their significant downstream effects. Wu et al. examined the role of MP-induced DNA damage to ovarian granulosa cells in mice after exposure to 100 mg/L of polystyrene microplastics (PSMPs) for 35 days [32]. In addition to noting increased reactive oxygen species (ROS) production, they found that the level of γH2AX, a protein DNA damage marker, was significantly higher in the PSMPs-exposed group versus the control. Comet assay analysis further substantiated their claims by demonstrating gradually increased comet tail length, another validated marker for DNA damage, in PSMPs-exposed cells. Ding et al. found similar results when exploring the size-dependent toxicity of PSMPs on mouse-derived gastric epithelial cells in showing that PSMPs exposure increased the levels of γH2AX and 8-OHdG, a marker of DNA single-strand breakage and oxidative stress, with smaller PSMPs sizes associated with more elevated levels of these biomarkers, compared to control [33].

### Endoplasmic Reticulum (ER)

MPs have been shown to induce stress on the endoplasmic reticulum (ER), a key organelle for protein synthesis, folding, and transport throughout the cell. Ragusa et al. examined human placental tissue for the presence of MPs and detected MP localization in the ER, consequently leading to ER dilation in the form of ballooning cristae and intracristal swelling [34]. With regards to the mechanism of action of MP-ER toxicity, Pan et al. demonstrated that oral administration of polystyrene microplastics (PS) activated ER-mediated hepatotoxicity, induced ROS development, and decreased mitochondrial membrane potential in mice via the eIF2α-ATF4-C/EBP homologous protein (CHOP) axis, which plays a critical role in cellular mitophagy and mitochondrial fission. Gene silencing of protein kinase RNA-like ER kinase (PERK), an ER stress sensor, was shown to significantly modulate these deleterious consequences of PS, in turn suggesting that PERK significantly impacts PS-induced cytotoxicity [35]. Wu et al. explored the effects of PSNPs on BEAS-2B mouse cells and bronchial epithelial cells that exist in humans as well. They found that PSNPs upregulated ER stress protein expression in IRE1α, PERK, XBP1S, and CHOP, while contributing to eventual cellular apoptosis and ferroptosis. Notably, these effects are reduced in the presence of ROS inhibitor N-acetylcysteine (NAC) [36]. MPs have been shown to lead to renal cell apoptosis in juvenile rats [37], hepatotoxicity in freshwater carp [38], and myocardial dysplasia and lung injury in chickens all through ER stress-induced inflammatory processes [39, 40], further highlighting not only the ER’s influence in mediating the harmful outcomes of MPs but also the need for further research in clinical models.

### Cell Membrane

Cell membranes are thought to work as a first shield for MPs entering the cell and altering the cellular environment. There are two possible mechanisms of MPs on cell membrane damage: direct damage to cell membranes or indirect damage to cell membrane structure by way of ROS. W Wang et al. revealed that polyethylene NPs caused cell membrane damage in a dose-dependent manner and that the particles easily permeated into lipid membranes, resulting in significant variations in dipalmitoyl phosphatidylcholine bilayer with lower density, fluidity changes, and membrane thickening [41]. JB Fleury et al. studied the physical effects of MPs on cell membranes and demonstrated that MPs adsorbed on lipid membranes considerably increase membrane tension even at low particle concentrations using experimental and theoretical approaches, resulting in a reduction of membrane lifetime [42]. As highlighted earlier, higher concentrations of MPs are reported to cause the production of more ROS in cells, leading to membrane damage indirectly [43]. MPs have been reported to be taken up by cells through several pathways: passive transport of cell membranes, diffusion assisted by transport proteins, and endocytosis [44]. After entering cells, MPs affect cells through internalization.

### Mitochondria

Following internalization, MPs are first localized to lysosomes and then transported to the mitochondria through the biofilm system, resulting in changes in mitochondrial membrane potential [45]. This leads to abnormal production of ROS at the bottom of the respiratory chain in the inner mitochondrial membrane, which in turn causes mitochondrial dysfunction to generate more ROS. Mitochondria are the primary site of damage, caused by the accumulation of NPs/MPs, leading generation of ROS in the cell [46]. R Trevisan showed the toxicity of PSNPs for mitochondrial energy production in developing zebrafish [47]. Y Gu et al. investigated the accumulation of different PSNPs (e.g., plain PS, carboxyl-functional PS-COOH, and amino-functional PS-NH2) at two particle sizes of 100 nm and 200 nm in the intestine and respiratory tree of Sea cucumber (*Apostichopus japonicus*), and found that the complex I, II, and III activities in the mitochondrial respiratory chain were significantly inhibited [48]. S Yang investigated the neurotoxicity of PSNPs on neural progenitor cells (NPCs) and hippocampal neurogenesis in a rodent model [49]. Accumulation of amine-modified polystyrene (PS-NH3 +) in NPCs caused mitochondrial dysfunction and energy depletion. 10 days of PS-NH3 + administration to C57BL/6 mice showed impaired hippocampal neurogenesis and memory retention. Li investigated the impacts of MPs on mitochondria in the human hepatocellular carcinoma (HepG2) cell line, showing that PSNPs treatment induced mitochondrial injuries, including morphological changes, decreased adenosine triphosphate production, and the loss of mitochondrial membrane potentials [50]. It resulted in increasing cell apoptosis, excessive ROS production, mitochondrial fission, and downregulating OPA1 and peroxisome proliferator-activated receptor- γ coactivator-1a proteins. This study showed a toxicity for the liver including the effect of mitochondrial-dependent apoptosis, ROS production, oxidative stress, mitochondrial morphological changes, and mitochondrial dynamics imbalance. RM Folgar et al. evaluated the altered molecular mechanisms on the human neural stem cell line (hNS1) after 4 days of exposure to 30 nm PSNPs (0.5, 2.5, and 10 μg/mL) and showed deficient COX5A expression causing mitochondrial dysfunction in skeletal muscle [51].

## Effects on Musculoskeletal System

### Cartilage/Growth Plate/Bone Growth

Regarding the effects of MPs on organs of the musculoskeletal system, bone-related effects have been the most studied. MPs have been shown to enter the bloodstream and reach the bone system [52, 53]. Sun et al. reported that 0.5 mg/d MPs exposure altered gene expression in mouse bone marrow cells enough to impact the hematological system [52]. While a 25-g mouse exposed by 0.5 mg/d for 28 days ingests 140 mg/kg per week, a 62 kg human was reported to ingest 80.64 mg/kg per week [54]. These numbers indicate that we cannot underestimate the impact of daily exposure to MPs on bone.

Several animal studies have shown that early MPs exposure can slow growth and that nanoparticle exposure can cause osteoporosis [55, 56]. Q Zhang et al. showed the possibility that MPs could induce ER stress and impair the endochondral ossification process [57]. Juvenile rats orally administered MPs for 28 days had shortened tibial length and sparse trabecular bone. In the growth plate, the thickness of the proliferative zone substantially reduced while the thickness of the hypertrophic zone increased significantly, and the chondrocytes were scarce and irregularly arranged. Pan et al. treated mice with PSMPs for 28 days and observed the accumulation of senescent osteoblasts in the bone trabecular following redundant skeletal growth and impaired trabecular bone microarchitecture [58]. MPs decreased the Ca/P ratio in serum, leading to bone resorption and disturbed bone reconstruction. They also found impaired autophagy with decreased autophagosome and reduced autophagy-related proteins in the senescent osteoblasts. The accumulation of senescent osteoblasts by MPs was eliminated by the re-establishment of autophagy by rapamycin.

I*n-vitro*, Giannandrea et al. assessed if NPs influenced murine bone cell cultures including pre-osteoblasts, osteocyte-like cells, and pre-osteoclasts [59]. In the study, NPs induced ROS production and apoptosis in various bone cell cultures and impaired the migration capability of pre-osteoblasts and the osteoclastogenesis of pre-osteoclasts. NPs also increased the expression of genes related to inflammatory pathways in pre-osteoblasts and osteocytes and disturbed the balance between osteoblastogenic and osteoclastogenic commitment.

In summary, with regard to bone growth related to cartilage, growth plate, and bone, the accumulation of MPs is shown to have a negative impact on healthy bone and periosteal development. Concerning arthritis, only a few studies have been conducted on rheumatoid-related issues [60], but we were unable to find any studies on the effects of MPs on bone and cartilage in osteoarthritis. Therefore, future research is needed to determine whether MPs accumulated in children could contribute to the development of osteoarthritis.

### Muscle

Despite the fact that many studies, mainly on marine species, have shown that MPs accumulate in liver and skeletal muscle in large amounts [61–64], there are very few studies on the effects of MPs on skeletal muscle. Chen et al. found MP contamination in the chicken skeletal muscles, which were directly collected from a large-scale chicken farm [65]. The PSMPs exposure inhibited energy and lipid metabolism and induced oxidative stress, a potential for neurotoxicity in the skeletal muscle. Primary myoblasts co-cultured with PSMPs in vitro showed that PSMPs exposure induced primary myoblast proliferation and apoptosis but decreased myoblast differentiation. Yang et al. investigated the effect of PSMPs on the skeletal muscle of piglets given diet supplementation with PSMPs [66]. Piglets in the 150 mg/kg PSMPs group exhibited decreased meat redness index and type I muscle fiber density and impaired muscle angiogenesis. Shengchen et al. showed that PSMPs exposure reduced the average cross-sectional area (CSA) and diameter of the muscle fibers, increased lipid deposition in tibialis anterior muscles of mice fed water containing 10 mg/L of 1–10 µm PSMPs or 10 mg/L of 50–100 µm PSMPs.

**Fig. 2:**
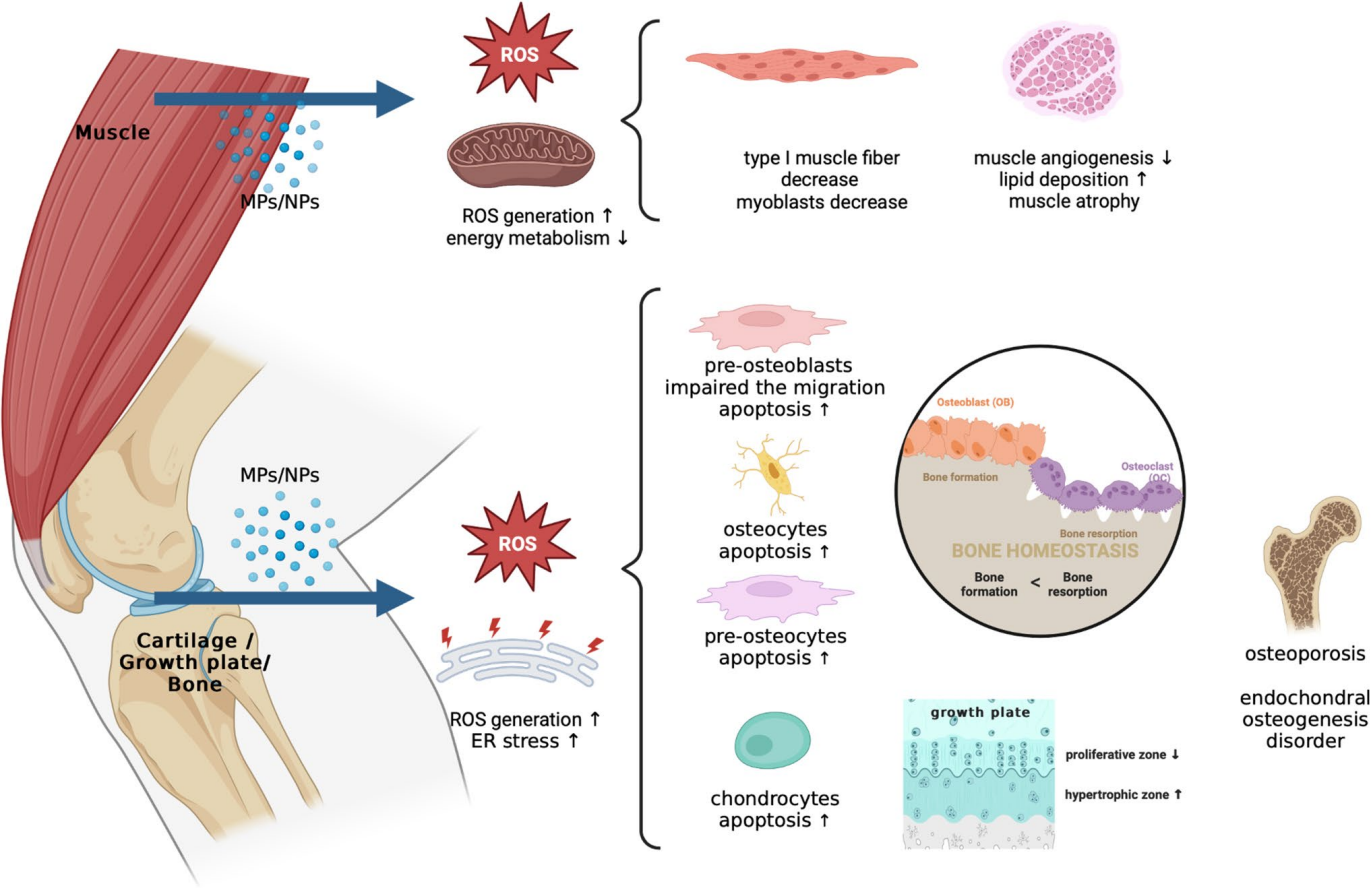
Effects of microplastics on musculoskeletal system, including the cartilage, growth plates, bones, and muscles. MPs = microplastics; NPs = nanoplastics; ROS = reactive oxygen species; ER = endoplasmic reticulum

The results of these studies on the effects of MPs on mammalian muscle consistently indicated harmful effects, highlighting the need for research involving human skeletal muscle. Additionally, no studies have been reported on ligaments or tendons, but it will be essential for understanding the overall impact of MPs on the musculoskeletal system (Fig. 2).

### Risk of Musculoskeletal Cancer

Due to the multidimensional effects of MPs—including the promotion of inflammatory states, the prevention of DNA regulation, and the activation of ROS—MPs have been associated with the development of cancer. Some MPs contain harmful chemicals and heavy metals, such as Mercury, Arsenic, and Aluminum, that are known carcinogens [67]. MPs have also been implicated in tumor progression, especially in colorectal cancer, through cell migration enhancement [68]. In addition to aiding in cell migration, MPs were also found to be associated with chemotherapy resistance in gastric cancer in mice through enhancement of ASGR2 in vivo [69]. These studies serve as examples to suggest MPs deleterious role in cancer risk and spread. There currently is no known association of MPs or NPs with sarcomas; as such, additional studies are needed to further explore the carcinogenicity of MPs, especially with musculoskeletal forms of malignancy.

## Future Directions

Considering the amount of exposure to microplastics over a lifetime, it is crucial to prioritize understanding the effects of MPs and NPs on children. In the outpatient setting, orthopedic surgeons should provide accurate information to the many parents who inquire about the effects of microplastics on their children’s musculoskeletal systems and how they should address these concerns. In modern daily life, it is extremely difficult to eliminate exposure to microplastics (MPs). They can enter the body through various means—oral ingestion via tableware and toys, or through body surfaces via helmets and medical devices. Therefore, to minimize the effects of MPs on the body, it is crucial to understand their nature and impacts thoroughly. This includes recognizing which organs are specifically affected by MPs and which are not. While the adverse effects on various human tissues, such as the heart and lungs, have been studied, research on the musculoskeletal system remains limited. Only recently has the accumulation of microplastics (MPs) in human bone marrow and synovium been confirmed in the orthopedic field [70, 71]. Although numerous animal studies have demonstrated the negative impact of MPs on bone function, there is a significant lack of research on muscles, tendons, and other connective tissues that interact with bone and control movement. Some authors believe that calcific tendinitis is related to the degenerative tear of the rotator cuff [72]. A foreign body such as MPs could potentially have an even greater impact on tendonitis, tendon rupture, and pain. A recent study has shown that MPs detected in the air during surgery increase more than usual [73]. In orthopedic surgery, polyethylene prosthetic components (e.g., plastic liners and inserts) and non-absorbable threads are occasionally used. Orthopedic surgeons should investigate disease-specific microplastic content in tissues, including muscles, tendons, and ligaments that can be collected during surgery, as well as conduct more research on the effects of the plastic implants they use.

## Conclusion

This review provided information on the latest research reports on microplastics, from basic concepts to their effects on the musculoskeletal system. Microplastics accumulation and deleterious effects on tissues have been reported in various organs, but there have been very few reports on the effects on the musculoskeletal system. At the laboratory level, microplastics were found to inhibit the healthy growth of periosteal cells and reduce muscle quality. This review highlights the need for investigations involving other parts of the musculoskeletal system and studies using human tissues at the disease level.

## Data Availability

No datasets were generated or analysed during the current study.
